# Long-Pulse Thermography Application for Detection and Localisation of Embedded Optical Fibres into Glass Fibre Composite

**DOI:** 10.3390/ma17246255

**Published:** 2024-12-21

**Authors:** Katarzyna Majewska, Magdalena Mieloszyk

**Affiliations:** Institute of Fluid-Flow Machinery, Polish Academy of Sciences, Fiszera 14, 80-231 Gdansk, Poland; mmieloszyk@imp.gda.pl

**Keywords:** pulsed infrared thermography, thermographic data processing, optical fibres, glass fibre composite, non-destructive testing

## Abstract

Composites have found applications in critical components and require a high degree of safety and reliability. To ensure this, structural health monitoring systems based on optical fibres embedded within structures are installed for continuous monitoring. Infrared thermography is a non-destructive method that can be applied to inspect the internal structure after manufacturing and during operation. This paper presents an application of pulsed thermography for observing and evaluating the internal structure of glass fibre-reinforced polymer samples with different arrangements of embedded optical fibres. The goal of the paper is to study the feasibility of using pulsed thermography to distinguish optical fibres from glass textile fibre bundles, as well as to track the arrangement of the optical fibres.

## 1. Introduction

Composite materials are widely utilised across numerous industries, including marine [1], aerospace [2], automotive [3], biomedical [4], and many others [5]. Their versatility and high-performance characteristics make them invaluable in these sectors. However, with their widespread use comes the necessity for rigorous safety standards. Any failure in composite structures can result in significant financial costs for repairs and pose risks of environmental damage, sometimes even leading to ecological catastrophes. This highlights the growing need to thoroughly understand their mechanical behaviour under various damage scenarios and to develop advanced non-destructive testing (NDT) methods for detecting and monitoring damage progression.

Ensuring the reliability of composites is essential for protecting both human lives and the environment. Damage in composite structures can originate in two primary ways: external impacts or internal failures. Internal failures may include issues such as delamination, matrix cracking, fibre-matrix debonding, or even the intrusion of water, dust, and contaminants during manufacturing. Additional challenges can arise from embedded elements like fingerprints, sensors, or other imperfections.

To address these challenges, structural health monitoring (SHM) systems have emerged as an effective solution. These systems, particularly those employing optical fibres (OFs), are designed to be permanently installed on or integrated into composite structures. By continuously tracking the integrity of the material, SHM systems provide early detection of damage, enabling timely interventions to prevent catastrophic failures and extend the lifespan of these materials. There are a lot of SHM systems; the most promising ones are these based on FBG sensors (e.g., buildings [6], bridges [7,8], wind turbine blades [9,10], ship hulls [11,12]), optical time-domain reflectometry, and Brillouin scattering methods [13,14]. OFs, with their diameters being slightly thicker than a human hair, can be embedded into a composite matrix [15,16]. Consequently, it is crucial to prevent failures when embedding an OF into a composite. Moreover, it is essential to have knowledge of the arrangement of these optical fibres within the elements to avoid damaging SHM system lines during tasks like the maintenance of real structures and constructions.

It is crucial to employ an NDT method for inspecting the internal structure of an element immediately after manufacturing and during its operational use. Infrared thermography (IRT) is an NDT technique that monitors heat distribution through vibration, ultrasonic excitation, and/or light impulse to assess the structural health of the analysed element [17]. IRT proves highly beneficial for diagnosing composite structures, including both glass fibre-reinforced polymers (GFRPs) and carbon fibre-reinforced polymers (CFRPs) [18,19]. Thermography methods can be categorised as passive and active [20]. Passive thermography involves the measurement of thermal variations in a material using an infrared vision device, without the need for external thermal sources. Unlike passive thermography, active thermography necessitates external heat sources to stimulate the materials under test. Common types of active thermography techniques include pulsed thermography (PT) in both transmission and reflection modes, stepped thermography (ST), lock-in thermography (LT), modulated thermography (MT), pulsed phase thermography (PPT), and frequency-modulated thermography (FMT) [21]. Optical-based excitation methods allow for structural excitation without affecting the temperature field of the structure, as is the case with contact-based transducers (ultrasound-based methods). In the case of PT, the choice of proper pulse parameters becomes a critical consideration. Typically, two types of excitation pulse shapes are used: rectangular and exponential [22,23]. Additionally, an approach based on frequency-modulated thermal excitation can be employed [24].

In PT, two distinct approaches are based on the relative placement of the excitation source and the infrared (IR) camera. These approaches include heat reflection, where the thermal excitation source and the IR camera are positioned on the same side of the sample, and heat transmission, where the thermal excitation source and the IR camera are located on opposite sides of the sample. PT is widely employed for damage detection in metallic structural elements, particularly in aerial structures [20], as well as in components made from CFRP [25] and GFRP composites [26]. In composite materials, IRT is invaluable for detecting various issues, including delamination [27], water ingress [28], matrix cracking [29], and thermal degradation [30].

**Figure 1:**
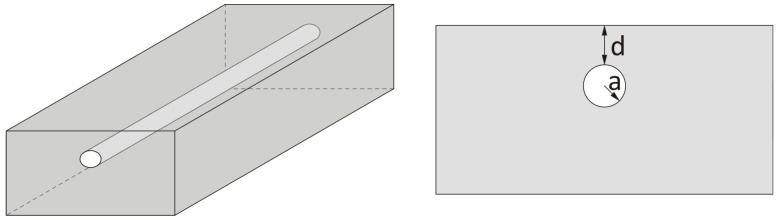
Geometry of semi-infinite material with included cylinder- or sphere-based object.

The current state of the art highlights the fundamental challenge of inspecting composite materials, which is driven by the increasing use of composite elements in our daily lives. The motivation behind this research stems from the lack of literature pertaining to the application of the modified long-pulsed thermography method for observing and evaluating the internal structure of GFRP samples with embedded OFs. While articles related to objects introduced in the form of cylinders and/or spheres under thermography techniques have been studied [31,32,33,34], they often involve different materials and structures, as well as varying dimensions (details in Figure 1 and Table 1). Furthermore, much of the current literature related to the detection of buried or embedded objects using IRT primarily focuses on military applications. These concealed objects often simulate buried mines, hidden, for instance, in sandy terrain.

**Figure 2:**
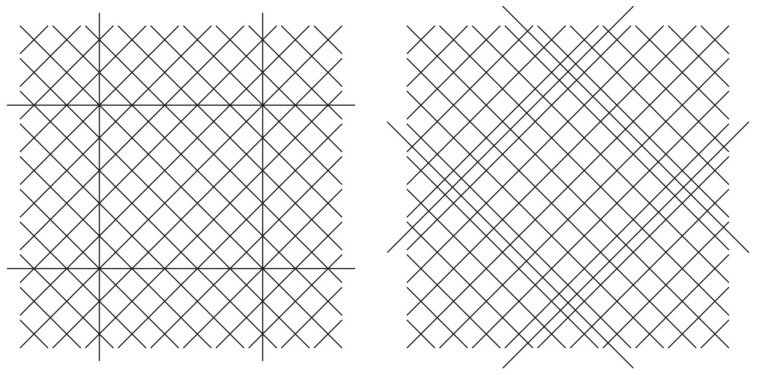
Mutual arrangement of the glass fibre bundle and optical fibres.

This paper presents an application of modified pulsed thermography for observing and evaluating the internal structure of four-layered GFRP samples with embedded OFs. These rectangular samples were manufactured using the infusion method, and each sample had a different arrangement of OFs. In every sample, four OFs were used. In the first case, the OFs were aligned parallel to the edges of the sample, denoted as ‘+’, while in the second case, they were oriented at a 45° to the edges of the sample, denoted as ‘x’—see Figure 2. Due to this, the primary aim of this study is to investigate the feasibility of using PT to distinguish OFs from the matrix fibre bundles and to track the arrangement of the OFs. Additionally, the paper analyses and establishes the optimal time and power of the light signal for PT.

The paper is organized as follows: firstly, GFRP samples with embedded OFs are described, and the manufacturing process is outlined. Next, the thermography imaging results for these samples are presented. Finally, a discussion of the achieved results and the limitations of the methods, as well as the conclusions, are presented.

## 2. Experimental Investigation

### 2.1. Samples Preparation

The laminates were fabricated using the vacuum infusion method. This process involved a combination of bidirectional textile (specifically, glass SGlass®) and epoxy resin. During the preparation process, the material was laid on a metal plate covered by PTFE (*polytetrafluoroethylene*) layer, allowing easy removal of the sample. An additional special layer covered the material plies for the purpose of an equal distribution of the resin during the manufacturing process. Due to this, the bottom surface of every sample was smooth, while the top surface was rough. A detailed description of the infusion process on a similar sample is explained in [36,37].

### 2.2. Samples and Setup

**Figure 3:**
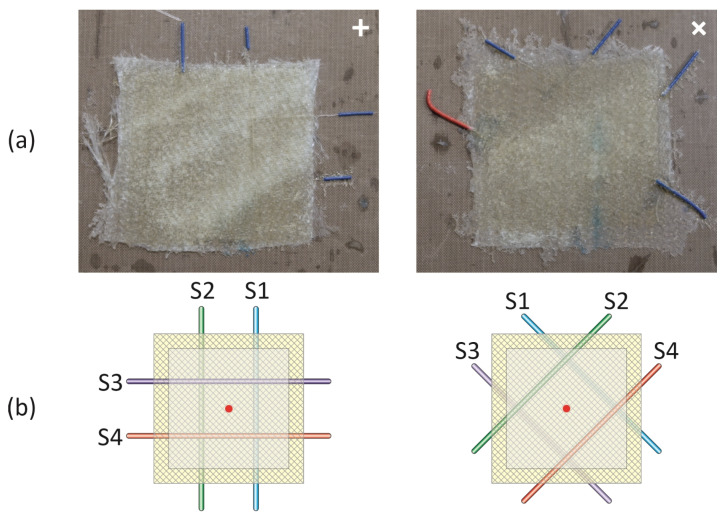

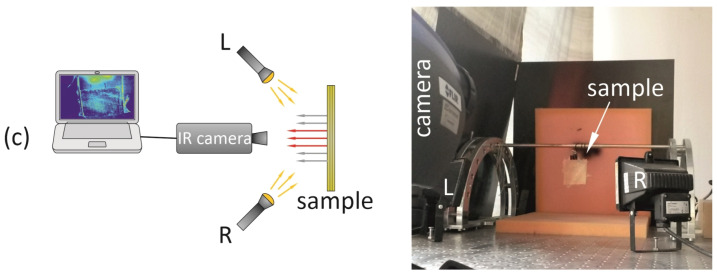
(**a**) Photos, (**b**) schemes of samples, (**c**) schema and photo of set-up.

The measurements were conducted on square GFRP samples, as depicted in Figure 3a. The dimensions of both samples were as follows: 60 mm × 60 mm × 1–2 mm. Each sample comprised four layers of composite material, with four embedded optical fibres (OFs). Two OFs were positioned between the 1st and 2nd layers, and the other two were placed between the 3rd and 4th layers counting from the bottom of the samples, as illustrated in Figure 3b. The physical properties of the glass material and the bare OFs are similar. The primary distinction lies in the fact that the glass fibres are intertwined to form a textile, while the OFs remain separate. The dimensions of the OFs are approximately 0.25 mm, which is smaller than the thickness of the textile (0.4 mm) used for a single layer of the composite sample. The internal structure of the prepared composite samples was examined using pulse PT in reflection mode, employing a thermal excitation source and an IR camera (FLIR SC-5600, FLIR Systems, Kent, UK) on the same side. The experimental setup is presented in Figure 3c. Measurements were carried out using two 150 W halogen lamps (identified as L and R), the IR camera operating at a measurement frequency of 100 Hz, a dedicated support frame, and a computer for camera software control. The light from the lamps induced changes in the temperature field of the samples at locations where any discontinuities, such as the OFs, were introduced.

### 2.3. Experiment Details

The primary objective of these experimental investigations was to assess the feasibility of utilising PT techniques to differentiate OFs from the matrix fibre bundles to trace the arrangement of the OFs within the sample and to determine the strength of the light signal. To achieve this, the following scenario of measurement was proposed. During the experiment, the smooth surface of the samples was stimulated using an optical source, consisting of two halogen lamps, while the infrared response was simultaneously observed on the same surface of the samples using an IR camera. Recording of the results initiated 5 s prior to the activation of the light source and stopped once the sample had cooled to ambient temperature. The light exposure lasted for 10 s. The introduction of light caused localised temperature variations in the sample.

To address these challenges, three measurement cases were considered for each sample (see Figure 3):

- Case B—using both synchronized lamps, both rated at 300 W;
- Case L—using the left lamp, rated at 150 W;
- Case R—using the right lamp, also rated at 150 W.

### 2.4. Samples Under Thermography Investigation

**Figure 4:**
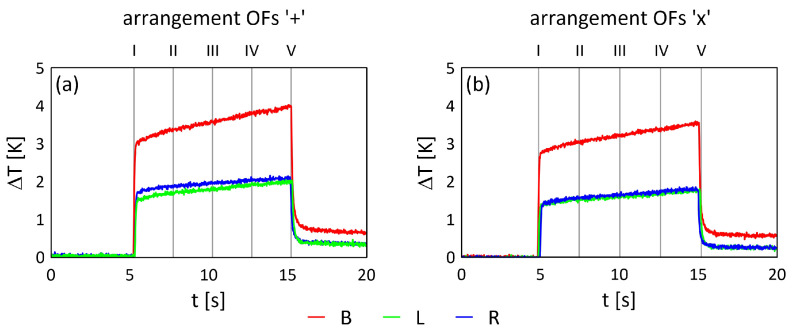
Timing graphs for two different arrangements of OFs with analysed frames (I–V): (**a**) arrangement OFs ‘+’, (**b**) arrangement OFs ‘x’.

Timing graphs for selected points (red dots) in the middle of each sample (Figure 3b) for the three exciting cases (B, L, R—as mentioned above) with marked analysed frames are presented in Figure 4. For the analysis, five specific frames were chosen, where ‘I’ denotes the moment heating started, and ‘V’ represents the moment when heating ended. It is evident that for both lamps in case B, the registered temperature changes (ΔT) are almost twice as high as for a single lamp (either L or R). The process of heating and cooling the samples is clearly visible and easy to observe.

The percentage difference of energy indicator ($I_{E}$) achieved by the sample during excitation with a single halogen lamp (either L or R) compared to using two halogen lamps is calculated using the following formula:

(1) $$I_{E}=100-\frac{case\;Y_{i}-case\;X_{i}}{case\;Y_{i}}*100$$

where *Y* = *B*, *X* = *L*, *R* and *i* = ‘+’, ‘x’ arrangement OFs.

The calculated percentage differences in energy indicator ($I_{E}$) are presented in Table 2. These differences are attributed to the asymmetric positioning of the lamps (L, R) with respect to the sample’s main axis. Specifically, lamp R was located closer to the sample, with a maximum distance of 30 mm, in comparison to lamp L. The selection of the measurement point (the red dot in the middle of the sample, as shown in Figure 3b) can also influence the observed results. From an experimental perspective, the middle point of the sample proved to be the most suitable, as it was free of OFs, ensuring an equal distribution of daylight and excitation light.

### 2.5. Typical Signal Filtering Method

In the field of infrared thermography, the most common post-processing techniques are based on either discrete Fourier transform or spatial derivatives of the thermal field. The first approach treats the values of each pixel over time as a 1D signal, while the second considers each thermogram as a separate image [38]. The discrete Fourier transform, despite being a time-consuming process, is an efficient method for identifying the location and depth of damage [39,40,41,42]. Damage depth determination relies on differences in phase. This method has been successfully employed in the analysis of steel plates with flat bottom holes (30 mm × 30 mm) at various depths, ranging from 1 mm to 4.5 mm [40,41]. Spatial derivatives of temperature values exhibit clear changes in proximity to defects. This technique has been used for detecting and localising cracks in CFRP specimens [38], as well as after pre-processing to reduce the influence of sample edges and for identifying different inclusion regions in thin GFRP samples [43].

In the cases presented, involving thin GFRP samples embedded with optical fibres (OFs), an analysis was conducted to assess the applicability of methods based on discrete Fourier transform and spatial derivatives of the thermal field. It is worth mentioning that these investigations focused on detecting and locating discontinuities in the form of OFs with a radius of approximately 0.125 mm, which were embedded in thin samples ranging in thickness from 1 mm to 2 mm. Furthermore, both the OFs and the sample fabric, composed of glass fibre textile, share very similar material properties. However, the discrete Fourier transform method, due to the strong similarities between these materials, did not reveal significant differences in phase. As a result, this method did not allow for the distinction of the studied regions. For the second method, which is based on the thermal field, the norm of the first spatial derivative of temperature was calculated using the relationship provided by [38]:

(2) $$DT\left(x,y,t\right)=\sqrt{{\left(\frac{\partial T}{\partial x}\right)}^{2}+{\left(\frac{\partial T}{\partial y}\right)}^{2}}$$

where *x* and *y* are coordinates in a sample plane.

Due to strong reflections from the sample’s edges, its texture, and the surrounding setup area, the method failed to accurately pinpoint the location of the OFs. After performing pre-processing, which involved subtracting the effects of the edges, texture, and surrounding influences from the sample’s response, the method was applied again. Unfortunately, even after this second attempt, the results did not meet the expectations. Given the unsatisfactory results obtained with conventional filtration techniques, the authors decided to develop their own filtration algorithm, as explained in detail in Section 2.6.

### 2.6. Proposed Signal Filtering Method

The filtering process is carried out within a defined area, specifically the internal section of the sample, as indicated by the grey rectangular region in Figure 3b. This approach effectively eliminates any potential influences from the background and sample edges. Given that the heating level of the composite material is influenced by the exposure time (Figure 4), the filtering process is applied individually to each frame.

The frame filtering process contains five main steps. Firstly, the frame is represented as a matrix, which is denoted as $F\left[i,j\right]=f_{ij}$ with size of *n* × *w* (number for cut off) equal to the number of pixels covered by the sample area. Then, a zero matrix $P^{c}\left[i,j\right]=p_{i,j}^{c}$ with the same size as the matrix *F* is created. Then, a following calculation procedure is performed separated for columns and rows of matrix *F*. For every column, it is described by a relationship:

(3) $$V_{kc}^{c}=max\left(F\left(i,j\right)\right)\;for\;kc=1,...,zc;\;zc\leq w;\;i=1,...,n;\;j=const$$

where *kc* means chosen part of a column. For enhanced sensitivity, it is assumed that there is an overlap of 25% (with a minimum of 7 elements) between the parts. The sizes of the divisions were determined based on the results of experimental investigations and the area influenced by the OFs, which was established as spanning five consecutive elements. During each step, the maximum value $V_{kc}^{c}$ is assigned to corresponding element in matrix $P^{c}$ at the same position as in the matrix *F*. An analogous procedure is performed for every column. As a result, a non-zero matrix $P^{c}$ is achieved.

A similar procedure is performed for rows of the matrix *F*. At the beginning, zero matrix $P^{r}\left[i,j\right]=p_{i,j}^{r}$ with the same size as the matrix *F* is created. The calculation procedure performed for every row can be presented by a relationship:

(4) $$V_{kr}^{r}=max\left(F\left(i,j\right)\right)\;for\;kr=1,...,zr;\;zr\leq n;\;j=1,...,w;\;i=const$$

where *kr* means chosen part of the rows. Similarly, like in the previous case, an overlap of the parts is assumed equal to 25%. In every step, the maximum value $V_{kr}^{r}$ is assigned to the corresponding element in matrix $P^{r}$ at the same position as in the matrix *F*. As a result, a non-zero matrix $P^{r}$ is achieved.

Maxima and their corresponding locations are determined independently for both rows and columns. This approach proves to be valuable for elements containing multiple embedded OFs with different arrangements relative to the sample’s coordinates. It is also effective for structures where the OFs are oriented either perpendicular or parallel to one of the sample’s axes, aligning with the rows or columns of the matrix *F*.

For samples where OFs are not aligned with any axis, we have $P^{c}=P^{r}$. Otherwise, $P^{c}\neq P^{r}$ because maxima are determined for areas with OFs influence only. Consequently, information about the occurrence of OFs is discretised, with density dependent on the length of parts *kr* or *kc*. To consolidate all available information in one location, matrices $P^{c}$ and $P^{r}$ are combined to create a single matrix P[i,j] using the following formula:

(5) $$\begin{array}{c} if\;p_{i,j}^{c}=p_{i,j}^{r}\\ then\;p_{i,j}=p_{i,j}^{c} \end{array}$$

otherwise

(6) $$\begin{array}{c} if\;p_{i,j}^{c}\neq p_{i,j}^{r}\\ if\;p_{i,j}^{c}>0\;then\;p_{i,j}=p_{i,j}^{c}\\ if\;p_{i,j}^{r}>0\;then\;p_{i,j}=p_{i,j}^{r} \end{array}$$

The experimental investigations allowed us to define ΔT levels that are referred to as raw material or OFs. This allowed for the determination of threshold values *m* dependent on excitation time. It is assumed that only values higher than the thresholds can be considered potential indicators of OFs.

Following this assumption, the matrix *P* is filtered, and

(7) $$if\;p_{i,j}<m\;then\;p_{i,j}=0$$

The procedure described above enables the identification of areas influenced by OFs. For enhanced clarity, the thickness of each OFs influence region is reduced to a single maximum value. Additionally, all non-zero values of matrix *P* are replaced by one, creating new matrix $Pz\left[i,j\right]=pz_{i,j}$. The procedure is described by the formula:

(8) $$if\;pz_{i,j}>0\;then\;pz_{i,j}=1\;for\;i=1,...,n;\;j=1,...,w$$

**Figure 5:**
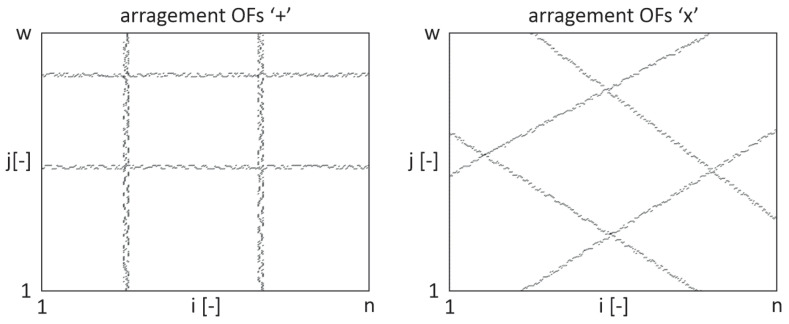
An example of OFs arrangement presented in the form of graphical visualisation of matrix *P*.

In Figure 5, two examples of achieved results for different OFs arrangements are presented.

## 3. Results

**Figure 6:**
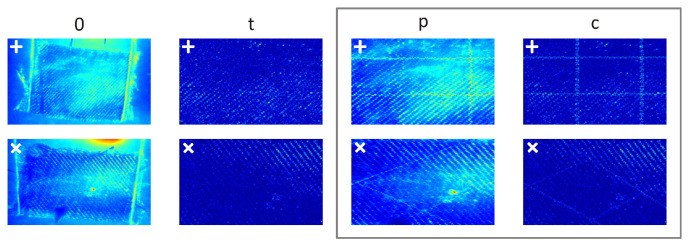
Exemplary frames: without ‘0’, after typical ‘t’, proposed ‘p’, and combination ‘c’ of typical and proposed signal filtering methods applied for both arrangements (‘+’, ‘x’) for case B (both lamps).

Figure 6 presents exemplary frames for the different filtering methods discussed in the paper. The pure thermograms of the sample, together with the fixing elements (see Figure 3c) and the background, are marked as ‘0’. In all the thermograms, the sample edges, sample surface pattern (glass fibre textile weaving), and setup elements are clearly visible. However, due to these factors, the locations of the OFs are only faintly marked or even invisible. The marks ‘t’, ‘p’, ‘c’ indicate zoomed areas that focus on the selected part of the sample (grey square in Figure 3b). The maps, denoted ‘t’, resulted from applying the typical signal filtering method, which involves spatial derivatives of the thermal field. This was done following signal pre-processing techniques, including subtracting the effects of edges, texture, and neighbourhood influences from the sample’s response. However, due to the similarity in material properties between the OFs and the sample fabric, the resulting maps remain unreadable, making it impossible to distinguish the regions of interest. The maps, denoted ‘p’, resulted from applying the proposed signal filtering method. This filtering approach proved to be a convenient and efficient solution, as it enabled the distinction of nearly all OFs and their arrangements, although it iss worth noting that the influence of the textile pattern was not completely removed. The maps, denoted ‘c’, resulted from the combination of both the proposed and typical signal filtering methods. This combination proved highly effective, allowing for the easy distinction of all OFs and their arrangements. Additionally, the complete removal of the textile pattern’s influence was achieved.

### 3.1. Sample with OFs Arrangement Denoted as ‘+’

**Figure 7:**
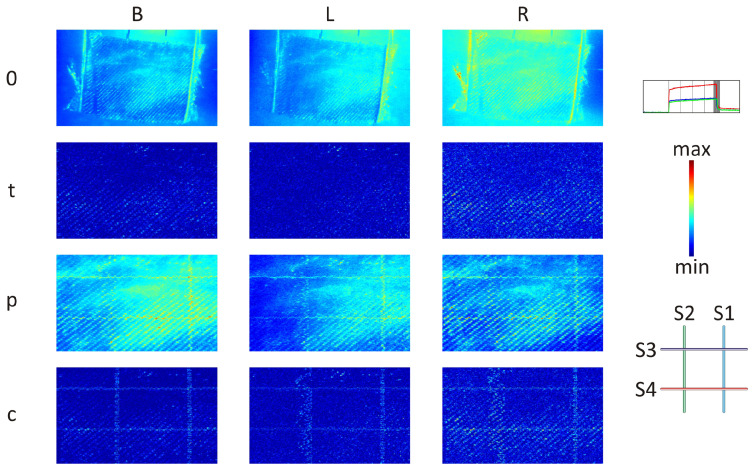
Analysed frame ‘V’: without ‘0’, after typical ‘t’, proposed ‘p’, and combination ‘c’ of typical and proposed signal filtering methods applied for case B (both lamps), L (left lamp), R (right lamp).

**Figure A1:**
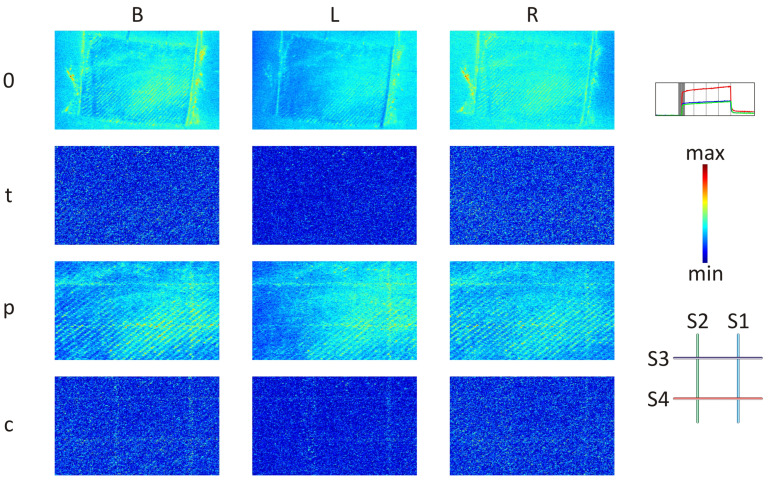
Analysed frame ‘I’: without ‘0’, after typical ‘t’, proposed ‘p’, and combination ‘c’ of typical and proposed signal filtering methods applied for case B (both lamps), L (left lamp), R (right lamp).

**Figure A2:**
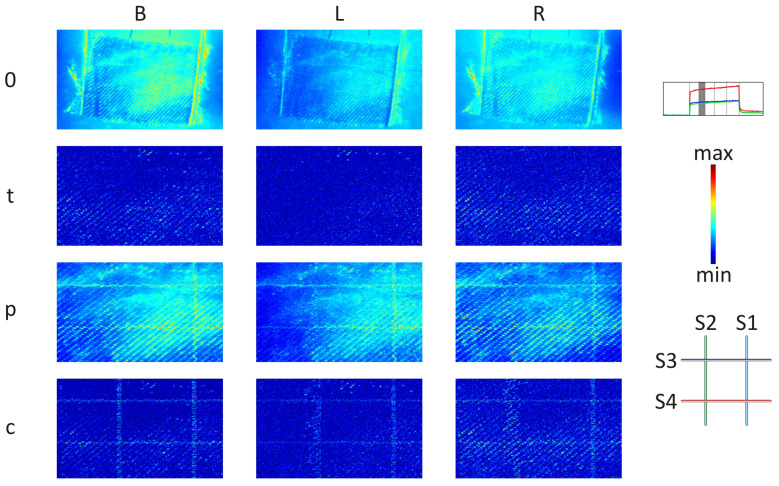
Analysed frame ‘II’: without ‘0’, after typical ‘t’, proposed ‘p’, and combination ‘c’ of typical and proposed signal filtering methods applied for case B (both lamps), L (left lamp), R (right lamp).

**Figure A3:**
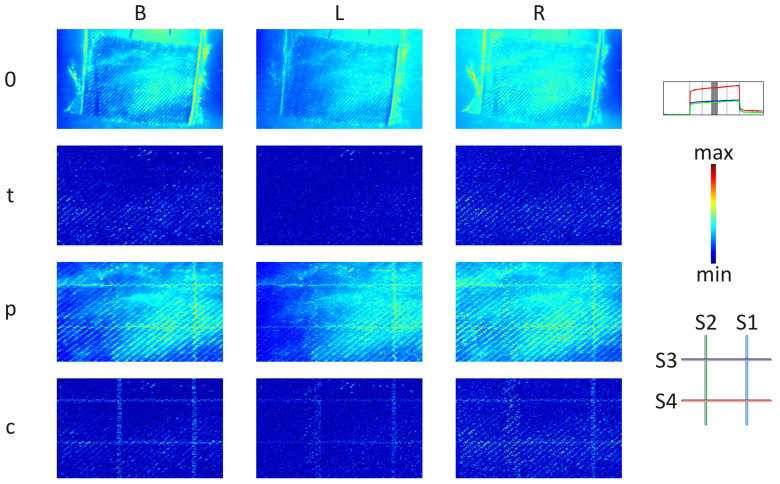
Analysed frame ‘III’: without ‘0’, after typical ‘t’, proposed ‘p’, and combination ‘c’ of typical and proposed signal filtering methods applied for case B (both lamps), L (left lamp), R (right lamp).

**Figure A4:**
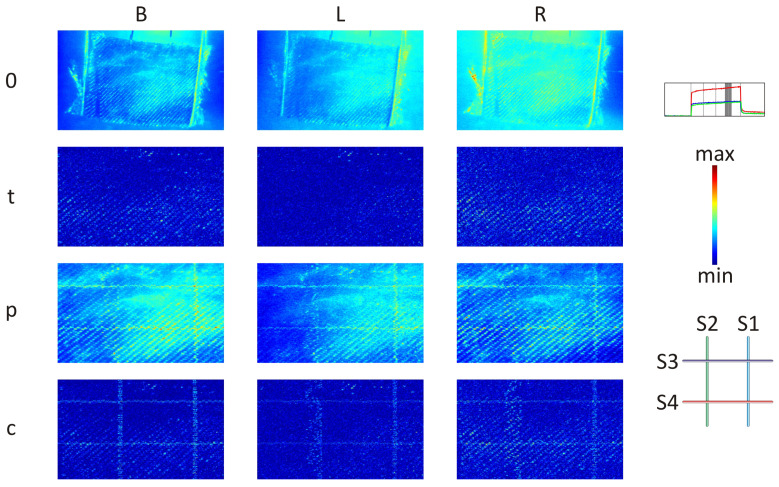
Analysed frame ‘IV’: without ‘0’, after typical ‘t’, proposed ‘p’, and combination ‘c’ of typical and proposed signal filtering methods applied for case B (both lamps), L (left lamp), R (right lamp).

The results are presented for frame ‘V’ (Figure 7), and the remaining frames can be found in the Appendix (Figure A1, Figure A2, Figure A3, and Figure A4). Contrary to the frames marked ‘II’–‘V’, in the case of frame ‘I’ (Figure A1), it is notably challenging to observe any changes in the maps. When comparing the three presented excitation cases (B, L, R) for frames ‘II’ to ‘V’, it becomes apparent that the OFs are most clearly visible in case L, where the influence of roughness (maps denoted ‘p’) was the lowest. This is primarily due to the amount of energy achieved by the sample and the uniformity of thermal excitation distribution on the sample’s surface. In cases B, the thermal energy distribution is relatively even on the surface, resulting in a similar roughness influence across the entire sample. Conversely, in cases B and R, the textile pattern is highly visible, making it challenging to completely eliminate and observe any further details (such as OFs or other discontinuities in the sample material) in maps denoted ‘p’. Consequently, cases L and R are considered the optimal choices for this purpose, since they facilitated the distinction of OFs without causing a significant excitation of sample roughness. The issue of sample roughness influence was addressed by employing a combination of both the proposed and typical signal filtering methods. This combination effectively revealed the localisation of the OFs and nearly eliminated the textile pattern (maps denoted ‘c’). Thanks to the equal distribution of excitation light provided by both halogen lamps and the successful removal of the textile pattern, case B emerged as the best choice. In all cases (B, L, and R), the application of the proposed signal filtering (maps denoted ‘p’) and a combination of both (combined) the proposed and typical (maps denoted ‘c’) signal filtering methods allowed for the differentiation of the OFs in the GFRP material. However, it remains challenging to determine the specific layers within which the OFs are located.

### 3.2. Sample with OFs Arrangement Denoted as ‘x’

**Figure 8:**
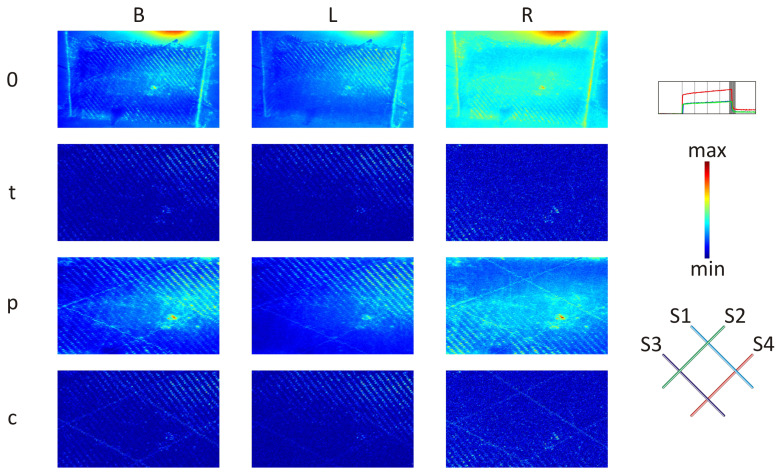
Analysed frame ‘V’: without ‘0’, after typical ‘t’, proposed ‘p’, and combination ‘c’ of typical and proposed signal filtering methods applied for case B (both lamps), L (left lamp), R (right lamp).

**Figure A5:**
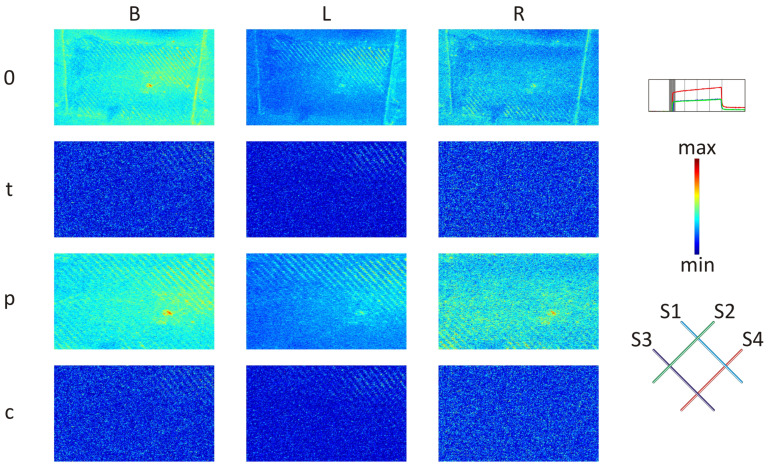
Analysed frame ‘I’: without ‘0’, after typical ‘t’, proposed ‘p’, and combination ‘c’ of typical and proposed signal filtering methods applied for case B (both lamps), L (left lamp), R (right lamp).

**Figure A6:**
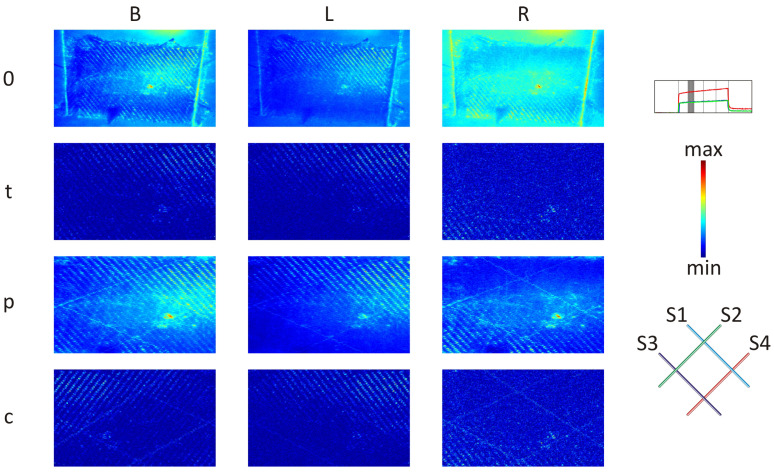
Analysed frame ‘II’: without ‘0’, after typical ‘t’, proposed ‘p’, and combination ‘c’ of typical and proposed signal filtering methods applied for case B (both lamps), L (left lamp), R (right lamp).

**Figure A7:**
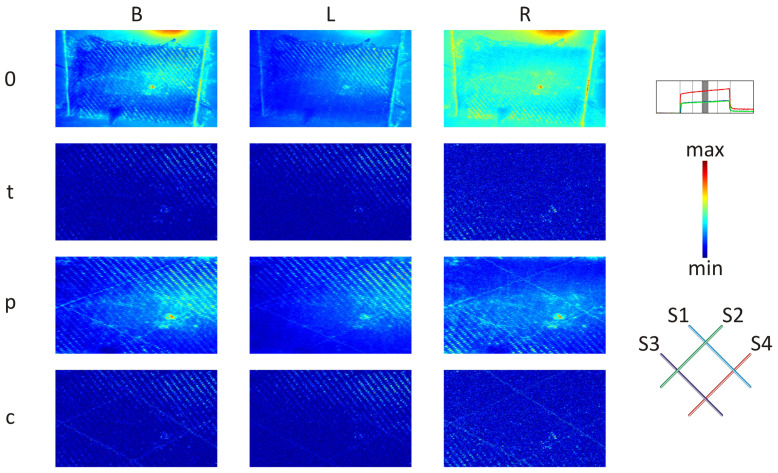
Analysed frame ‘III’: without ‘0’, after typical ‘t’, proposed ‘p’, and combination ‘c’ of typical and proposed signal filtering methods applied for case B (both lamps), L (left lamp), R (right lamp).

**Figure A8:**
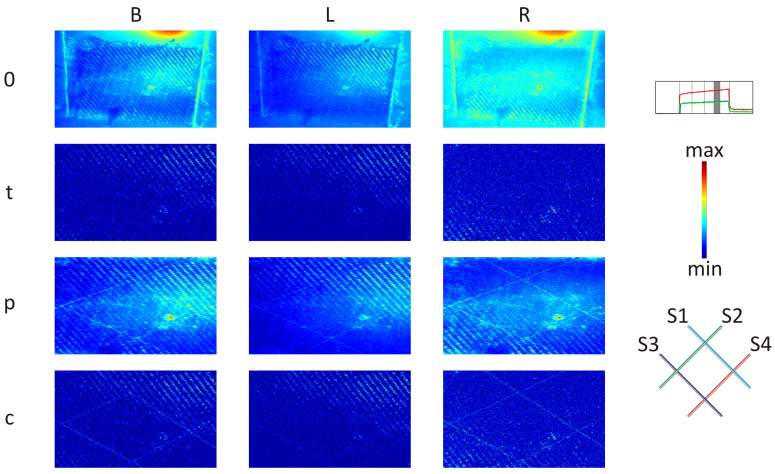
Analysed frame ‘IV’: without ‘0’, after typical ‘t’, proposed ‘p’, and combination ‘c’ of typical and proposed signal filtering methods applied for case B (both lamps), L (left lamp), R (right lamp).

The results are presented for frame ‘V’ (Figure 8), and the remaining frames can be found in the Appendix (Figure A5, Figure A6, Figure A7, and Figure A8). Contrary to the frames marked ‘II’–‘V’, in frame ‘I’, it is the hardest to see any differences in the sample’s surface. Comparing three presented excitation cases (B, L, and R) for the II–V frames, it is possible to observe that the OFs are likely to be distinguished in the B and R cases (maps denoted ‘p’), where the roughness influence was the lowest. For the L cases, the sample’s surface roughness had the most substantial influence on the thermal energy distribution. Due to this, the OFs effect has been almost neglected. It looks like the sample was not in the middle between the halogen lamps. The light coming from the R lamp covered the whole sample’s surface, while the L one’s upper-right quarter of the sample can be observed as a strong excitation (cyan). Despite this, in the B case, the textile pattern is also visible, and the arrangement of OFs is possible to be determined (maps denoted ‘p’).

Also for this sample for all cases (B, L, and R) after applying the proposed signal filtering (maps denoted ‘p’), as well as the combination of both the proposed and typical signal filtering (maps denoted ‘c’) methods, it is possible to distinguish the OFs in the GFRP material, but it is impossible to determine between which layers the OFs are located.

## 4. Discussion

**Figure 9:**
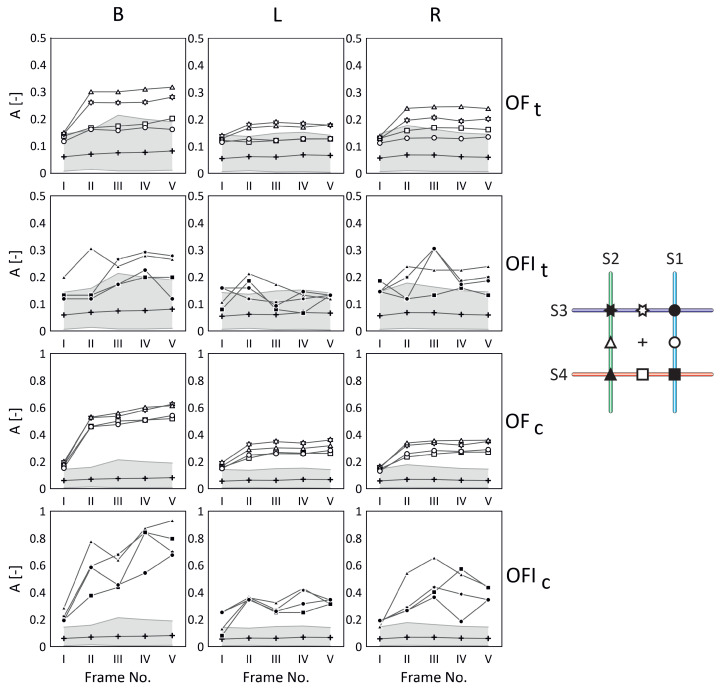
Comparison of the results for ‘+’ case.

**Figure 10:**
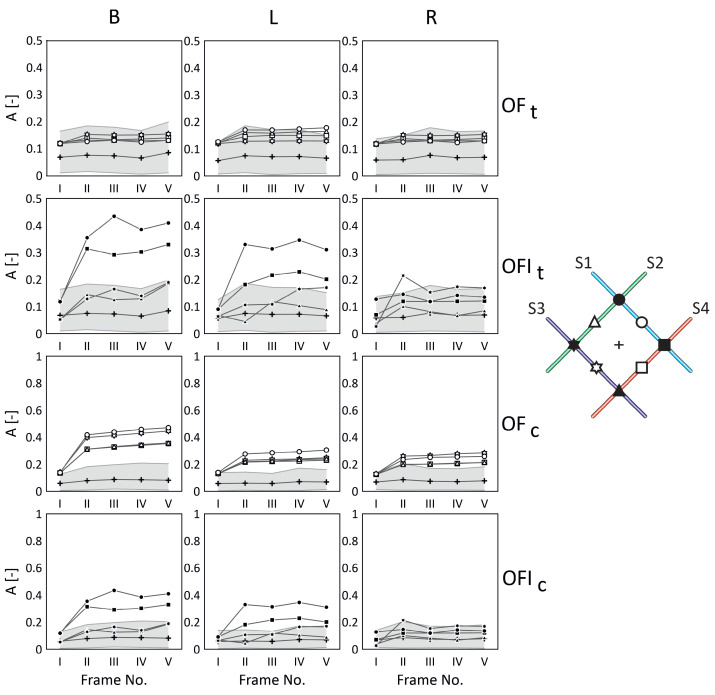
Comparison of the results for ‘x’ case.

In Figure 9 and Figure 10, the obtained results are presented for the two discussed cases of the fibre-optic arrangements, ‘+’ and ‘x’, relative to the arrangement of the glass fibre bundle, respectively—compare these with Figure 2.

In both figures, in the columns, the measurement cases related to the lamp light exposure (B, L, and R) are presented. Moreover, the first two rows present the results for the typical ‘t’ method, while the next two rows show the results for the combination ‘c’ method (the typical and proposed approaches), where OF relates to the results for optical fibre, and OFI relates to the intersection of optical fibre. On the right-hand side of the figures, for clarity and reference, the studied optical fibre arrangements are shown, together with the optical fibre numbers and the marks used during the analysis for each optical fibre (OF) and their intersections (OFI). The small cross in the middle represents the response of the pure material. It was calculated as an average value for the responses within a defined 9 × 9 evenly distributed area surrounding the measurement point (cross), and the final result was obtained by averaging these in relation to each frame from ‘I’ to ‘V’. Additionally, the grey area represents the maximum material response value in relation to each frame from ‘I’ to ‘V’.

In both cases (‘+’ and ‘x’), the best results were achieved for a combination of both the proposed and typical signal filtering methods (marked as ${\mathrm{OF}}_{c}$/${\mathrm{OFI}}_{c}$ in Figure 9 and Figure 10). However, it is easy to notice that the trace of optical fibres is less visible in the case of the sample with its arrangement denoted as ‘x’ than it is in the case of the sample with its arrangement denoted as ‘+’. That is due to the mutual arrangement of the optical fibre and glass fibre textile stacking sequences. In both samples, the textile stacking sequence is [−45/45/−45/45], the optical fibre arrangement is different, and the problem of visibility is presented schematically in Figure 2. Contrary to the sample with its arrangement denoted as ‘+’, in the case with its arrangement denoted as ‘x’, the optical fibres are hidden in the glass textile bundles. Also, it is visible that in both cases, the responses from the optical fibres and their intersections are stronger than the average value of the pure material (cross). However, the method ‘c’ (combination of both the proposed ‘p’ and typical ‘t’ ones) allowed us to achieve the results clearly visible outside the grey areas (Figure 9 and Figure 10) for case ‘+’ and for most of the results for case ‘x’.

For a better understanding, examine the results presented in Table 3. Comparing the results for the typical ‘t’ method (${\mathrm{OF}}_{t}$) and the combination ‘c’ (${\mathrm{OF}}_{c}$) signal filtering method for the optical fibres (marked S1, S2, S3, and S4), it is observed that in the case of both arrangements, ‘+’ and ’x’, all the optical fibres are clearly visible outside the grey areas (Figure 9 and Figure 10) for the combined ‘c’ method. Also, in the case of optical fibres intersections, in the case ‘+’, the combination ‘c’ of methods allowed us to observe all the intersections, and for case ‘x’, the results are very similar for both methods.

## 5. Conclusions

This paper explored the use of modified pulsed thermography (PT) to examine the internal structure of glass fibre-reinforced polymer (GFRP) samples with embedded optical fibres (OFs). The goal of the study was to assess how effective PT is in detecting and distinguishing OFs from the surrounding fibre bundles in GFRP composites.

Typical filtering techniques, such as Fourier transforms and spatial derivatives, struggled to distinguish the optical fibres from the surrounding material. This was mainly due to the material and geometric similarity of the optical and glass fibres, as well as external experimental conditions that interfered with the thermal signal. To solve this, the authors developed a new filtering algorithm that isolates the internal regions of the sample and analyses the thermal responses more effectively. The proposed methodology can reliably separate the OFs from the rest of the material.

The research focused on two different arrangements of OFs (‘+’ and ‘x’) and tested various filtering methods to help in tracking and identifying the OFs. In the ‘+’ configuration, the OFs and their intersections were clearly visible and consistently appeared outside the grey area representing the material’s thermal response. However, in the ‘x’ configuration, the visibility of the OFs was reduced due to their positioning within the glass textile bundles. Despite this, the combined filtering method (‘c’) still outperformed the typical ‘t’ method alone in terms of fibre detection.

Three things are worth mentioning that the research was carried out for:

- Objects with dimensions:
  – An optical fibre—ca 0.125 mm in radius;
  – A sample—1–2 mm in thickness;
- OFs and GFRP samples—These are made out of almost the same materials;
- Four OFs embedded into the GFRP sample—These constitute less than 0.5% of the volume fraction of the sample.

The authors’ method offers a promising tool for the non-destructive evaluation and structural health monitoring of composite materials. However, challenges related to sample roughness, manufacturing precision, and environmental conditions still need to be addressed. One major issue was the roughness of the sample surface, particularly the pattern of the glass fibre textile, which affected the clarity of the thermal images. The number and positioning of the halogen lamps used for excitation had to be adjusted depending on the specific sample. The distance from the light source to the sample, as well as the angle of light delivery, were crucial factors for obtaining clear results. The precision of the manufacturing process was also a concern. Unevenness in the resin distribution within the composite could affect the thermal response, leading to less-accurate results. Additionally, environmental factors such as daylight were shown to influence the results. The experiments, conducted on both sunny and cloudy days, demonstrated that external light could interfere with the thermal measurements. However, the combined filtering methods proved effective in reducing these environmental influences.

Although the method is highly effective at identifying the position and arrangement of the fibres and their intersections, it is not suitable for determining the exact object’s depth within the material (sample height).

The authors wish to continue their studies to overcome the problems that have arisen.

## Figures and Tables

**Table 1:** Comparison of introduced/embedded objects parameters.

No.	Sample	Object (Cylinder- or Sphere-Based)					Ref.
	**Material**	**Method**	**Material & Form**	**Radius** **a [mm]**	**Distance d [mm] from Surface**	**Orientation According to Surface**	
1	graphite	drilled	air gap	0.25	0.16	parallel	[33]
2	epoxy	embedded	SS rod	0.25	0.11	parallel	[33]
3	epoxy	drilled &	air gap &	0.25	0.11	parallel	[33]
		filled with	SS rod				
4	epoxy	drilled	air gap	2.50	0.45–0.55	not parallel	[31]
				6.35			
5	GFRP	embedded	resin rod	2.55	15.00	parallel	[32]
				1.25			
6	soil	erosion	air gap	∼245.92	50.08	not parallel	[34]
				∼253.25			
				∼255.52			
				∼273.09			
7	wood	embedded	Mylar sheet	5.00	0.30–0.80	parallel	[35]
				28.00 × 4.00	0.80–0.90		
				8.00	>1.40		
				14.00 × 3.00	>2.00		
8	GFRP	embedded	silica glass fibre	0.125	0.25	parallel	here

SS—stainless steel.

**Table 2:** Differences in energy indicator $I_{E}$ [%].

OFs Arrangement	L	R
‘+’	49.2	51.5
‘x’	52.4	49.5

**Table 3:** Comparison of the visibility of optical fibres and their intersections for the selected frame ‘V’.

		Frame ‘V’	
		**‘+’**	**‘x’**
	B	S2, S3, S4	non
${\mathrm{OF}}_{t}$	L	S2, S3	S1, S2
	R	S2, S3, S4	non
	B	S1–S4, S2–S3, S2–S4	S1–S2, S1–S4
${\mathrm{OFI}}_{t}$	L	non	S1-S2, S1–S4, S2–S3
	R	S1–S3, S2–S3, S2–S4	non
	B	all	all
${\mathrm{OF}}_{c}$	L	all	all
	R	all	all
	B	all	S1–S2, S1–S4
${\mathrm{OFI}}_{c}$	L	all	S1–S2, S1–S4
	R	all	non

## Data Availability

The original contributions presented in this study are included in the article. Further inquiries can be directed to the corresponding author.
